# MGL ligand expression is correlated to BRAF mutation and associated with poor survival of stage III colon cancer patients

**DOI:** 10.18632/oncotarget.4495

**Published:** 2015-07-02

**Authors:** Kristiaan Lenos, Jeroen A.C.M. Goos, Ilona M. Vuist, Sjoerd H. den Uil, Pien M. Delis-van Diemen, Eric J.Th. Belt, Hein B.A.C. Stockmann, Herman Bril, Meike de Wit, Beatriz Carvalho, Susan Giblett, Catrin A. Pritchard, Gerrit A. Meijer, Yvette van Kooyk, Remond J.A. Fijneman, Sandra J. van Vliet

**Affiliations:** ^1^ Department of Molecular Cell Biology and Immunology, VU University Medical Center, Amsterdam, The Netherlands; ^2^ Current address: Laboratory of Experimental Oncology and Radiobiology, Center for Experimental Molecular Medicine, Academic Medical Center, Amsterdam, The Netherlands; ^3^ Department of Pathology, VU University Medical Center, Amsterdam, The Netherlands; ^4^ Current address: Swammerdam Institute for Life Sciences, University of Amsterdam, Amsterdam, The Netherlands; ^5^ Department of Surgery, Spaarne Gasthuis, Haarlem, The Netherlands; ^6^ Current address: Netherlands Cancer Institute, Amsterdam, The Netherlands; ^7^ Department of Surgery, Erasmus Medical Center, Rotterdam, The Netherlands; ^8^ Department of Pathology, Spaarne Gasthuis, Haarlem, The Netherlands; ^9^ Department of Medical Oncology, VU University Medical Center Amsterdam, The Netherlands; ^10^ Department of Biochemistry, University of Leicester, Leicester, UK

**Keywords:** colorectal cancer, BRAF, glycosylation, MGL, C-type lectin

## Abstract

Colorectal cancer (CRC) is the third most prevalent cancer type worldwide with a mortality rate of approximately 50%. Elevated cell-surface expression of truncated carbohydrate structures such as Tn antigen (GalNAcα-Ser/Thr) is frequently observed during tumor progression. We have previously demonstrated that the C-type lectin macrophage galactose-type lectin (MGL), expressed by human antigen presenting cells, can distinguish healthy tissue from CRC through its specific recognition of Tn antigen. Both MGL binding and oncogenic BRAF mutations have been implicated in establishing an immunosuppressive microenvironment. Here we aimed to evaluate whether MGL ligand expression has prognostic value and whether this was correlated to BRAF^V600E^ mutation status. Using a cohort of 386 colon cancer patients we demonstrate that high MGL binding to stage III tumors is associated with poor disease-free survival, independent of microsatellite instability or adjuvant chemotherapy. *In vitro* studies using CRC cell lines showed an association between MGL ligand expression and the presence of BRAF^V600E^. Administration of specific BRAF^V600E^ inhibitors resulted in decreased expression of MGL-binding glycans. Moreover, a positive correlation between induction of BRAF^V600E^ and MGL binding to epithelial cells of the gastrointestinal tract was found *in vivo* using an inducible BRAF^V600E^ mouse model. We conclude that the BRAF^V600E^ mutation induces MGL ligand expression, thereby providing a direct link between oncogenic transformation and aberrant expression of immunosuppressive glycans. The strong prognostic value of MGL ligands in stage III colon cancer patients, *i.e*. when tumor cells disseminate to lymph nodes, further supports the putative immune evasive role of MGL ligands in metastatic disease.

## INTRODUCTION

Colorectal cancer (CRC) is the third most common cancer worldwide and a major cause of cancer deaths [1]. Development of CRC is accompanied by genomic instability, either chromosomal instability or microsatellite instability (MSI), which occurs in approximately 85% and 15% of primary tumors, respectively [2]. MSI tumors tend to have a relatively good prognosis, exemplified by the fact that only 3% of CRC metastases display MSI. This is probably due to the generation of immunogenic neo-antigens by MSI positive tumors, which induce the attraction of tumor-infiltrating lymphocytes with anti-tumor activity [3]. However, once tumors succeed to metastasize, MSI positive tumors have an extremely poor prognosis [4]. Interestingly, while BRAF^V600E^ mutations occur in about 10% of CRC cases [2] and are an important negative prognostic biomarker for CRC [4–6], the frequency of BRAF mutations among metastatic MSI tumors is much higher (approximately 35%) than among chromosomal instable tumors (<5%) [2]. These data imply that escape from immune evasion is crucial in tumor metastasis formation, and suggest that BRAF mutations may play an important role in this process.

The immune system plays a crucial, yet double-edged role in cancer progression, whereby tumor-promoting inflammation and the avoidance of immune destruction are now considered to be emerging hallmarks of cancer [7]. Immunosurveillance is important in controlling tumor growth and indeed the intra-tumoral infiltration of memory-type CD8^+^ T cells is a clear predictor of patient survival [8]. However, cancer cells deploy several immune evasion strategies to avoid eradication by the immune system, including the secretion of immunosuppressive factors or the recruitment of regulatory T cells (Tregs) or myeloid-derived suppressor cells. Currently, oncogenic alterations are increasingly linked to immune (dys)function during cancer progression. For instance, in melanoma the BRAF^V600E^ mutation mediates immune suppression through an increase in numbers and fitness of inhibitory immune cells, which can be alleviated through the use of specific BRAF inhibitors [9].

The occurrence of tumor-associated glycans plays a so far underappreciated role in tumor progression and the role of the immune system herein. We hypothesize that tumor-associated glycans could actively contribute to immune evasion by the tumor. Development of adenocarcinomas is frequently accompanied by changes in the glycosylation pattern of tumor cells, illustrated by a two-fold reduction in total glycosylation [10]. In many epithelial cancers the expression of truncated *O*-glycans, such as Tn antigen (GalNAcα-Ser/Thr), are predominating and these have been associated to bad prognosis, metastasis and poor survival [11]. Exposed Tn antigens are ligands for the Ca^2+^-dependent C-type lectin receptor MGL (macrophage galactose-type lectin/CD301/CLEC10A) [12], which is mainly expressed on immature dendritic cells (DCs) and macrophages [13, 14]. The carbohydrate specificity of MGL overlaps with the binding specificity of the snail lectin *Helix Pomatia* Agglutinin (HPA), which is highly specific for Tn antigen and has been associated with metastasis formation [15, 16]. Previously, we reported in a small group of CRC patients that MGL specifically recognizes the tumor-derived mucin MUC1 via binding to the Tn antigen [17]. The fact that cancer-associated aberrant *O*-glycans influence antigen processing and immune responses when taken up by DCs [18] suggests a dynamic interplay between aberrant glycosylation on tumor cells and the immune system.

The MGL receptor is upregulated on so-called tolerogenic human DCs and macrophages cultured in the presence of corticosteroids [19]. Furthermore, MGL also interacts with effector T cells, resulting in reduced proliferation, cytokine secretion and the induction of T cell apoptosis [14]. Engagement of MGL by tumor-associated Tn antigen leads to activation of the ERK-CREB signaling pathway and a robust production of the anti-inflammatory cytokine IL-10 [20]. These MGL-triggered DCs promote the differentiation of Tregs [21]. Collectively, the elevated expression of MGL on tolerogenic antigen presenting cells [19] and preferential recognition of tumor-derived glycans suggests that MGL-positive antigen presenting cells can enhance tumor progression.

We recently found that MGL ligand expression on T cells is regulated via the MAPK pathway [22], suggesting a possible role for activating BRAF^V600E^ mutations in the aberrant Tn antigen expression on tumor cells. In the present study we aimed to evaluate whether MGL ligand expression has prognostic value and whether its expression was correlated to BRAF^V600E^ mutation status. Our results demonstrate a direct regulation of tumor cell glycosylation by BRAF^V600E^ both *in vitro* and *in vivo*. Moreover, using a well characterized series of 386 colon cancer patients [23, 24], we now show for the first time that high expression of MGL ligands is an independent prognostic marker for stage III CRC patients, resulting in a significantly worse CRC-specific survival and higher recurrence rate.

## RESULTS

### High MGL ligand expression in stage III colon cancer patients is associated with decreased disease-free and CRC-specific survival

**Figure 1:**
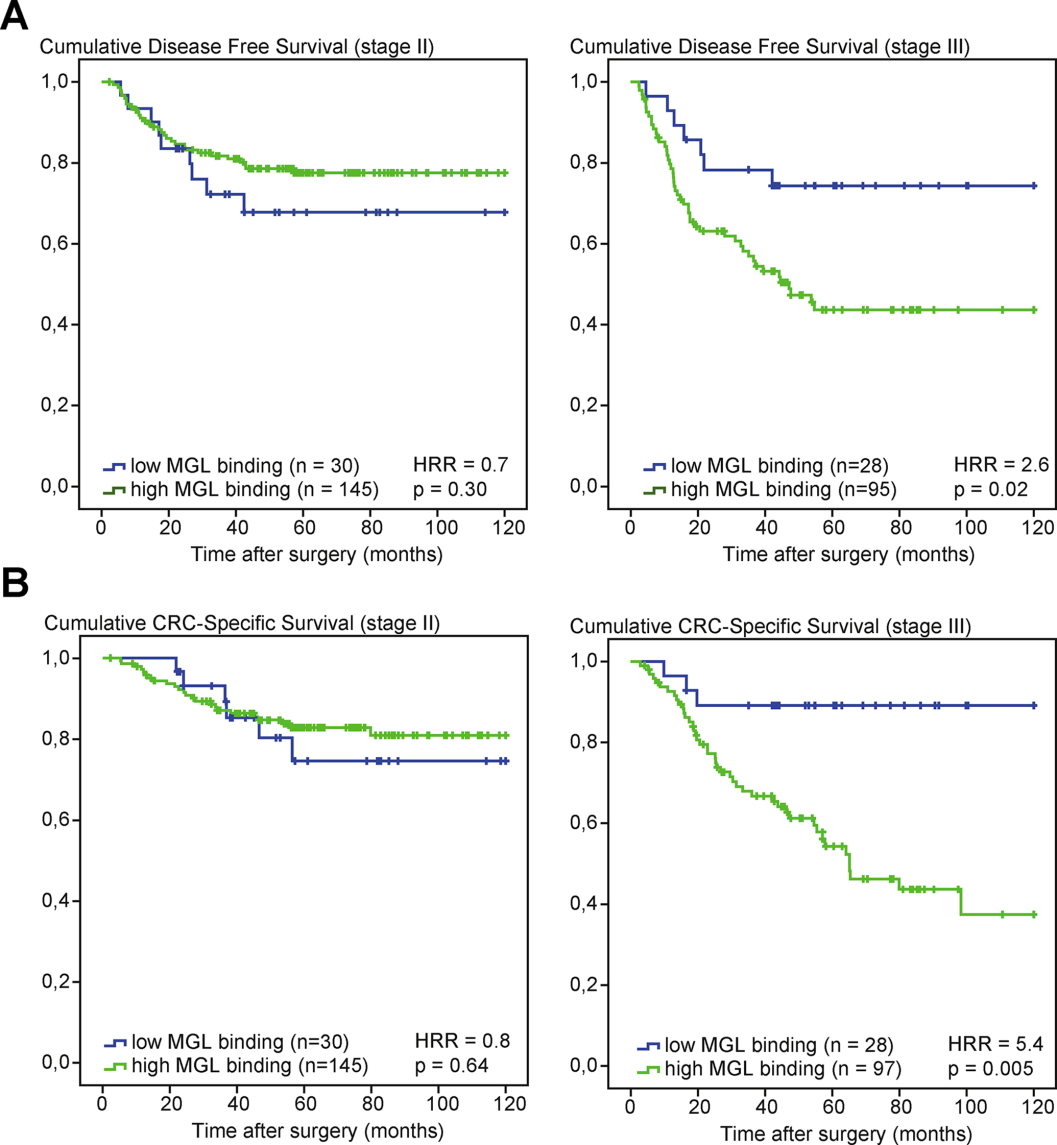
High MGL ligand expression in stage III CRC patients is associated with poor survival **A.** Kaplan–Meier curves of disease-free survival (DFS) of stage II (left panel) and stage III (right panel) colon cancer patients according to MGL ligand expression. **B.** Kaplan–Meier curves of cancer-specific survival (CSS) of stage II (left panel) and stage III (right panel) colon cancer patients according to MGL ligand expression. Patients deceased within 3 months after surgery were excluded from analysis. HRRs and *p*-values were determined by Cox regression analysis.

Based on prior findings that the C-type lectin MGL can distinguish between the healthy and tumor-derived mucin MUC1 [17] and its immunosuppressive role in T cell immunity [20, 21], we aimed to assess the prognostic value of MGL ligand binding in a series of 386 well characterized stage II and stage III colon cancer patients [23]. Tissue microarrays (TMAs) were stained for MGL ligand expression (Supplementary Figure 1) and scored for intensity and frequency of staining in the cytoplasm of neoplastic cells. Whereas no differences in survival rates between low and high MGL binders were observed in the stage II patient population, clear differences were apparent when only stage III patients were considered, with a CRC-specific 10-year survival (CSS) rate of 89.3% for low MGL-binders *versus* 54.6% for high MGL-binders (*p* = 0.02, Table 1). Moreover, high MGL-binding stage III patients had a higher recurrence rate than low-MGL binding patients (51% and 25% respectively), resulting in a much shorter disease free survival (DFS) for these patients (median = 32.0 months (high MGL-binders) *versus* 48.0 months (low MGL-binders)) (Table 1). Indeed, Cox regression demonstrated a significant association of MGL-binding with DFS (Hazard risk ratio (HRR) 2.6; *p* = 0.02; 95%CI 1.2–5.7) (Figure 1A) and CSS (HRR 5.4; *p* = 0.005; 95%CI 1.2–17.4) for stage III patients (Figure 1B). Stepwise backward Cox regression analysis, including age, location of tumor, differentiation grade and angioinvasion, demonstrated the strength of MGL-binding as an independent prognostic factor for CSS in stage III colon cancer patients (HRR 4.3; *p* = 0.02; 95% CI 1.3–14.0).

**Table 1:** Comparison of clinical data specified for MGL binding and disease stage

Characteristic		Low MGL binders						High MGL binders					
		Total		Stage II		Stage III		Total		Stage II		Stage III	
		N	%	N	%	N	%	N	%	N	%	N	%
	N (total)	58	100	30	100	28	100	243	100	145	100	98	100
Stage	II	30	51.7	30	100	0	0	145	59.7	145	100	0	0
	III	28	48.3	0	0	28	100	98	40.3	0	0	98	100
	MSS	40	83.3	21	91.3	19	76.0	181	84.6	101	84.2	80	85.1
	MSI	8	16.7	2	8.7	6	24.0	33	15.4	19	15.8	14	14.9
	unknown	10		7		3		29		25		4	
	mucinous	12	20.7	7	23.3	5	17.9	49	20.2	28	19.3	21	21.4
	non-mucinous	46	79.3	23	76.7	23	82.1	194	79.8	117	80.7	77	78.6
	ulcererous	45	77.6	22	73.3	23	82.1	184	75.7	108	74.5	76	77.6
	non-ulcererous	13	22.4	8	26.7	5	17.9	59	24.3	37	25.5	22	22.4
	angioinvasion	8	13.8	2	6.7	6	21.4	49	20.2	12	8.3	37	37.8
	No angioinvasion	50	86.2	28	93.3	22	78.6	194	79.8	133	91.7	61	62.2
	chemo	16	27.6	1	3.3	15	53.6	77	31.7	23	15.9	54	55.1
	no chemo	42	72.4	29	96.7	13	46.4	166	68.3	122	84.1	44	44.9
	recurrence	16	28.1	9	31.0	7	25.0	81	33.6	31	21.5	50	51.5
	no recurrence	41	71.9	20	69.0	21	75.0	158	65.6	112	77.8	46	47.4
	unknown	1		1		0		2		1		1	
Diff grade	bad	3	5.2	1	3.3	2	7.1	28	11.5	12	8.3	16	16.3
	moderate	53	91.4	28	93.3	25	89.3	197	81.1	120	82.8	77	78.6
	good	2	3.4	1	3.3	1	3.6	18	7.4	13	9.0	5	5.1
Tumor stage	T1	1	1.7	0	0	1	3.6	3	1.2	0	0	3	3.1
	T2	4	6.9	0	0	4	14.3	12	4.9	0	0	12	12.2
	T3	50	86.2	28	93.3	22	78.6	202	83.1	127	87.6	75	76.5
	T4	3	5.2	2	6.7	1	3.6	26	10.7	18	12.4	8	8.2
Nodal stage	N0	30	51.7	30	100	0	0	145	59.7	145	100	0	0
	N1	19	32.8	0	0	19	67.9	64	26.3	0	0	64	65.3
	N2	9	15.5	0	0	9	32.1	34	14.0	0	0	34	34.7
No. of nodes examined	mean ± SD	9.1 ± 4.6		7.6 ± 4.0		10.6 ± 4.8		8.7 ± 4.8		8.4 ± 4.8		9.2 ± 4.9	
Sex	male	31	53.4	11	36.7	20	71.4	130	53.5	79	54.5	51	52.0
	female	27	46.6	19	63.3	8	28.6	113	46.5	66	45.5	47	48.0
Age (years)	mean ± SD	70.1 ± 12.5		72.1 ± 9.9		68.0 ± 14.7		70.6 ± 11.9		71.0 ± 12.1		70.0 ± 11.8	
	Median (range)	70.8 (34.5–90.0)		71.0 (54.3–90.0)		70.6 (34.5–87.3)		72.8 (28.5–92.1)		73.6 (28.5–92.1)		72.6 (37.2.5–91.8)	
Follow up	mean ± SD	61.8 ± 32.2		62 ± 34.2		61.6 ± 30.7		56.9 ± 33.3		63.4 ± 32.3		47.4 ± 32.6	
	Median (range)	54.8 (9.9–142.6)		52.3 (21.9–129.2)		57.7 (9.9–142.6)		55.7 (2.27–148.6)		59.7 (2.27.5–139.6)		44.3(2.8–148.6)	
DFS	mean ± SD	53± 34.3		52.9 ± 36.6		53.1 ± 32.3		50.6 ± 35.0		58.9 ± 34.0		38.3 ± 33.2	
	Median (range)	43.8(4.5–142.6)		42.4(5.5–129.2)		48.0(4.5–142.6)		47.4 (2.27–148.6)		57.3(2.27.5–139.6)		32.0(2.27.5–148.6)	
Deceased	deceased	23	39.7	14	46.7	11	39.3	118	48.6	57	39.3	61	62.2
	not deceased	35	60.3	16	53.3	17	60.7	125	51.4	88	60.7	37	37.8
CRC-specific death*	deceased	9	15.5	6	20.0	3	10.7	68	28.1	24	16.6	44	45.4
	not deceased	49	84.5	24	80.0	25.0	89.3	174	71.9	121	83.4	53	54.6
	missing	0		0		0		1		0		1	

* In stage III patients the CRC-specific death is significantly higher for high MGL binders compared to the low MGL binders. *P* < 0.02, as determined by Pearson's Chi-square test. Abbreviations: chemo, adjuvant chemotherapy, MSS, microsatellite stable, MSI, microsatellite instability, diff, differentiation, DFS, disease-free survival, CRC, colorectal cancer

### Prognostic value of MGL binding is independent of MSI status or adjuvant chemotherapy

**Figure 2:**
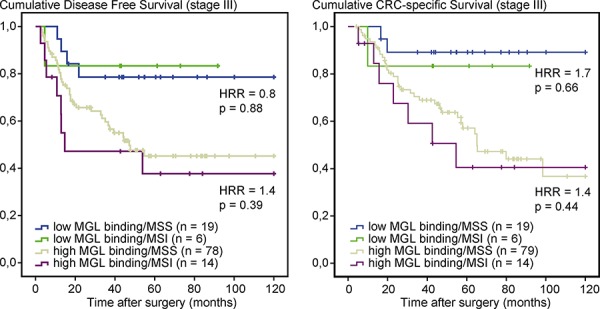
MGL-mFc binding is independent of MSI status in stage III CRC patients Kaplan–Meier curves of disease-free Survival (DFS) and cancer-specific survival (CSS) curves in stage III colon cancer patients, stratified for MSI status and MGL-binding. Patients deceased within 3 months after surgery were excluded from the analysis. HRRs and *p*-values comparing MSS to MSI within low or high MGL-binding groups were determined by Cox regression analysis.

MSI-positive early stage CRCs have been associated with a relatively good prognosis [25]. To evaluate whether MGL ligand expression is associated with MSI or microsatellite stability (MSS), stage III CRC patients were stratified for MSI status and MGL-binding. No significant differences were observed in DFS or CSS between patients with MSS or MSI tumors in either the low MGL-binding or in the high MGL-binding group (Figure 2). Stratification of stage III CRC patients for treatment with adjuvant chemotherapy also revealed no significant differences in DFS and CSS in either the low or high MGL-binding group (Supplementary Figure 2). In summary, our results indicate that MGL ligand expression is a prognostic biomarker in stage III colon cancer patients, irrespective of MSI status or adjuvant chemotherapy consistent with the putative immunosuppressive effects of aberrant glycosylation.

### Presence of BRAF^V600E^ mutation is associated with high expression of MGL-binding ligands

**Figure 3:**
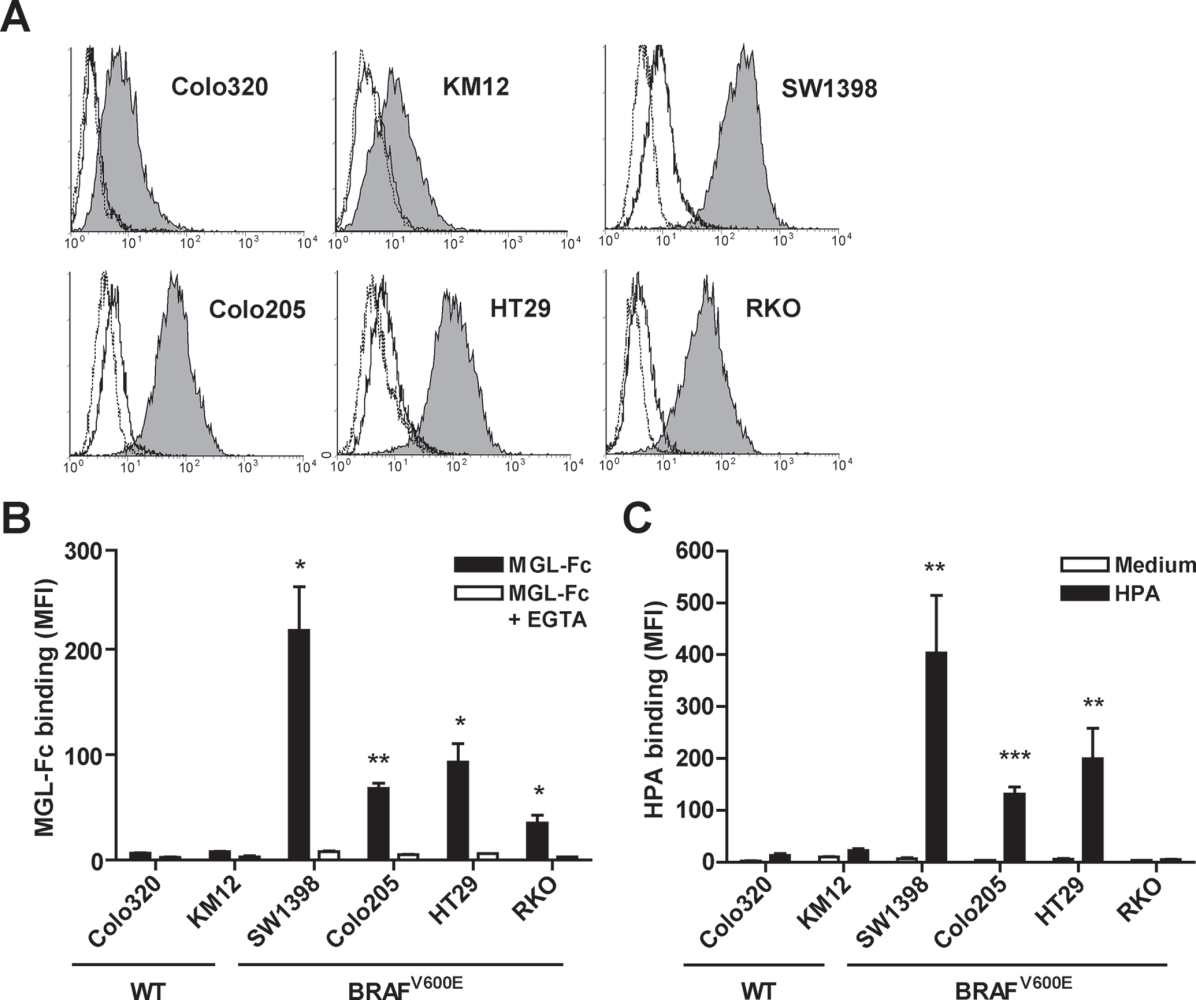
High expression of MGL ligands was associated with presence of the BRAF^V600E^ mutation A panel of colorectal cancer cell lines was analyzed for MGL-Fc and HPA binding using flow cytometry. Cells were harvested at 70–80% confluency. Representative flow cytometry plots are shown in **A.** Filled histograms indicates MGL-Fc binding, solid line represents MGL-Fc binding in presence of EGTA and dotted black line represents the secondary antibody control. **B, C.** Combined flow cytometric data of three independent experiments. Mutational status of *BRAF* is depicted below the graph. Black bars represent the MGL-Fc (B) and HPA (C) staining (Mean Fluorescent Intensity, MFI), white bars represent MGL-Fc binding in the presence of EGTA (B) or in case of HPA the medium control (C) *p < 0.05; **p < 0.01; ***p < 0.005.

To investigate the relationship between *BRAF* mutations and aberrant glycosylation we first turned to an *in vitro* approach using a panel of well characterized CRC cell lines. Confounding effects of *KRAS* and *EGFR* were avoided by selecting cell lines that lacked mutations or amplifications in these genes. The *BRAF* and MSI status of these cell lines are shown in Table 2. To examine the expression of tumor-specific carbohydrate ligands, especially Tn antigen, the binding of MGL-Fc [12] was investigated by flow cytometry. The *BRAF* wild-type cell lines Colo320 and KM12 had a very low expression of MGL ligands, whereas a high amount of MGL-Fc bound to the cells bearing the *BRAF^V600E^* mutation (Figure 3A and 3B). All binding was MGL-specific, as shown by the complete abrogation of binding in the presence of the Ca^2+^-chelator EGTA. With the exception of RKO, high MGL-Fc binding is accompanied by high binding of the Tn antigen-specific lectin isolated from *Helix pomatia* (*Helix pomatia* agglutinin, HPA, Figure 3C) [26]. No associations were found with MSI status. Overall, we found a clear difference in MGL ligand and thus tumor-associated glycan expression between cells that were wild-type for *BRAF* and those that harbor the BRAF^V600E^ mutation.

**Table 2:** Mutations in CRC related genes in panel of CRC cell lines

Cell line	BRAF	MSI/MSS
Colo320	WT	MSS
KM12	WT	MSI
SW1398	V600E	N.A.
Colo205	V600E	MSS
HT29	V600E	MSS
RKO	V600E	MSI

All cell lines contain wild type KRAS and EGFR.

Abbreviations: N.A., not available; WT, wild type

### Inhibition of BRAF^V600E^ reduces MGL ligand binding *in vitro*

**Figure 4:**
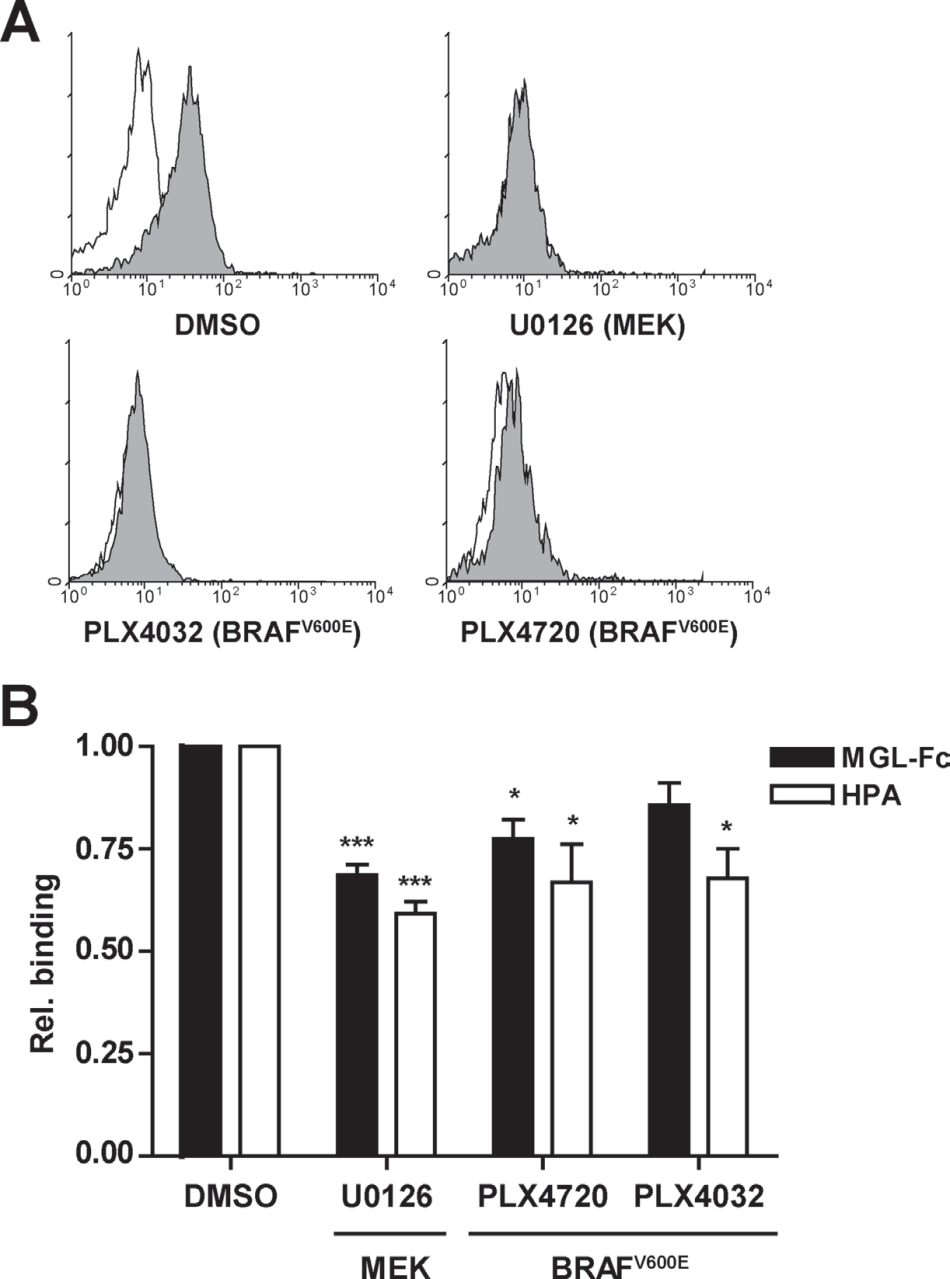
Inhibition of the MAPK pathway reduces MGL ligand binding HT29 cells were either mock- (DMSO) treated or treated with the MEK inhibitor (U0126) or the BRAF^V600E^ inhibitors PLX4032 (Vemurafenib) or PLX4720 (25 μM each). **A.** After a 4 hr treatment, HT29 cells were analyzed for intracellular phospho-ERK1/2 levels by flow cytometry. Representative flow cytometry plots are shown. Filled histograms indicates the phospho-ERK1/2 staining, solid line represents the isotype control. **B.** HT29 cells were incubated for 24 hrs with the inhibitors and analyzed for MGL-Fc and HPA-binding using flow cytometry. MGL-binding in the presence of EGTA was used to normalize the signal, HPA-binding was normalized to the negative control, binding is displayed as relative binding compared to mock-treated cells. Data are combined results of four independent experiments.

Since we observed high expression of MGL ligands in cells harboring activating mutations in *BRAF*, we next assessed whether interference with *BRAF^V600E^* and the downstream MAPK pathway would revert expression of MGL ligands on the tumor cell surface. Therefore the *BRAF* mutant cell line HT29, which expresses high levels of MGL ligands, was treated with the selective BRAF^V600E^ inhibitors PLX4032 (Vemurafenib) and PLX4720 and a specific inhibitor of MEK (U0126), which acts downstream of BRAF to phosphorylate ERK1/2. As expected, phosphorylated ERK levels strongly decreased upon BRAF^V600E^ or MEK inhibition, confirming the efficacy of inhibitor treatment (Figure 4A). Indeed, inhibition of BRAF or MEK activity resulted in decreased binding of MGL-Fc and HPA, suggesting a decreased expression of Tn antigens (Figure 4B).

### Induction of BRAF^V600E^ increases MGL ligand binding *in vivo*

**Figure 5:**
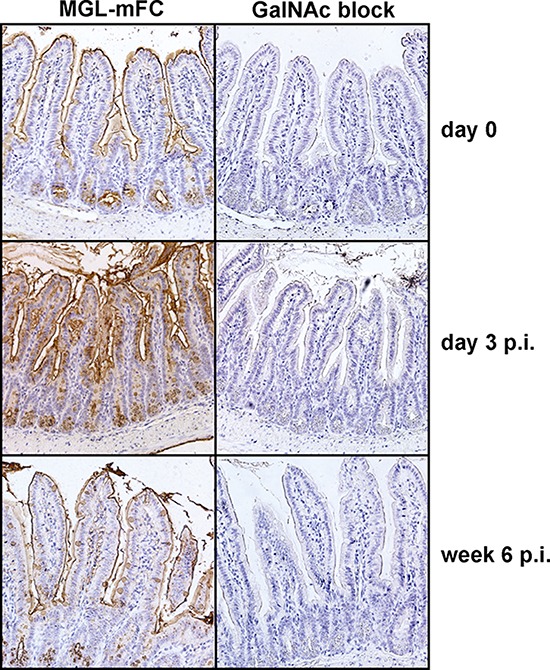
*In vivo* expression of BRAF^V600E^ results in increased MGL ligand expression Intestinal tissue material of the previously described BRAF^V600E^ inducible mouse model [27] was used to perform immunohistochemistry. Mice were sacrificed at day 0, 3 or 6 weeks post-induction (p.i.) of BRAF^V600E^ and stained with MGL-mFc, in the presence or absence of free GalNAc monosaccharides, as a control for MGL-specific binding to glycan structures present in the tissue, followed by DAB and haematoxylin staining. Pictures were taken with a Nikon E800 light microscope, at 200x magnifications.

To confirm the *in vitro* findings *in vivo*, we made use of a previously described inducible mouse model in which BRAF^V600E^ can be specifically induced from the endogenous promoter in proliferating cells of the gastro-intestinal tract [27]. In this model phosphorylation of ERK1/2 was increased three days after induction of mutant BRAF, followed by a p16^INK4a^-induced senescent phenotype [27]. We examined MGL-mFc binding to intestinal epithelial cells three days after BRAF^V600E^ induction and demonstrate that this binding is strongly increased (Figure 5). Although some staining of the mucus layer is apparent at day 0 and six weeks post induction, the epithelial cells lack intracellular MGL-binding glycans. In contrast, clear intracellular staining of the epithelial cells is visible at day three post induction, demonstrating an increased production of MGL-binding glycans by the cells at that time point. No staining was observed in the presence of an excess amount of free GalNAc ligands, demonstrating the specificity of the MGL binding. MGL binding was reduced to basal levels at later time points (six weeks post-induction), coinciding with the onset of senescence and a decrease in phospho-ERK levels [27]. These data show that oncogenic activation of *BRAF in vivo* is sufficient to drive MGL ligand expression.

## DISCUSSION

Here, we show for the first time a correlation between oncogenic activation of BRAF and the altered glycosylation that is often observed in CRC. Inducible activation of BRAF^V600E^ mutation *in vivo* directly resulted in increased expression of MGL ligands, whereas *in vitro* inhibition of BRAF^V600E^ and its downstream target MEK reduced this expression, indicating that BRAF mutation positively correlates to MGL-binding. Furthermore, we demonstrate that expression of MGL ligands is associated with poor survival and disease recurrence, in particular in stage III but not in stage II colon cancer patients, independent of MSI status or treatment with adjuvant chemotherapy. Therefore, the specific binding of MGL to tumors expressing these aberrantly glycosylated antigens has the potential to be used as a novel prognostic biomarker for stage III colon cancer patients.

The Tn antigen, one of the preferred ligands of MGL, has previously been associated with worse survival [11] and recent research indicates that tumor-specific Tn expression not only promotes tumor cell invasiveness [28], but also alters the immunogenicity of tumor antigens [29]. The tumor microenvironment in CRC is generally regarded as immunosuppressive, whereby a lower number and decreased maturation stage of infiltrating DCs is associated with a worse disease prognosis [30]. Interestingly, the prognostic value of MGL-binding to tumor cells is predominantly evident in stage III colon cancer patients and not in stage II patients, i.e. when tumor cells are no longer confined to the intestine but have spread into the local lymph-nodes. Here they come into contact with the peripheral immune system where they can interact with DCs outside the microenvironment of the primary tumor. We postulate that the lymph node MGL-positive DC subsets might very effectively suppress immune responses [31] through the secretion of high levels of immunosuppressive cytokines, such as IL-10 [20], and the induction of regulatory T-cell responses [21], thereby supporting immune evasion of tumor cells. The escalation in cancer immune escape could directly contribute to a worse prognosis of CRC patients, since after surgery remaining dormant tumor cells may not be effectively removed by the immune system.

Although we demonstrate a correlation between *BRAF* mutation and upregulation of specific carbohydrate ligands for the C-type lectin MGL, there is an apparent discrepancy between the high amount of 76% of patients that carry MGL-binding glycans (Figures 1 and 2) and the estimated incidence of BRAF mutations in approximately 10% of CRC patients. Considering that the *in vitro* inhibition of the BRAF downstream target MEK also affected MGL ligand expression, we assume that activating mutations in members of the MAPK pathway other than BRAF, such as KRAS and EGFR, may also cause aberrations in tumor cell glycosylation. In support of this hypothesis, high MGL-binding in breast carcinoma was recently associated with the expression of HER2/neu [32], which is an upstream activator of the MAPK-cascade and has been related to an immunosuppressive tumor microenvironment [33]. These findings suggest that various oncogenic alterations involved in activation of the MAPK pathway may participate in modulation of the tumor-related glycome. As such, the present study merely emphasizes the need to further investigate the exact relationship between activation of the MAPK pathway, alterations in glycosylation, and its effects on immunosuppression in various types of cancer.

The downstream targets of the activated MAPK pathway that regulate the glycosylation changes are not clear yet. Defects in the glycosylation enzymes T-synthase and Cosmc were shown to result in high expression of Tn antigen and have been implicated in several types of cancers [34], although others have reported that these mutations are very rare in human cancer, including CRC [35]. The aberrant glycosylation might also result from changes in the enzymes responsible for the initial *O*-glycosylation steps, the polypeptide:GalNAc transferases (pp:GNT's) [36]. Some of these transferases are overexpressed or mutated in cancer [37–41]. In addition, Gill et al. demonstrated that the tumor-specific relocalization of pp:GNT's from the golgi to the ER resulted in high density of truncated *O*-glycosylation and elevated Tn levels on mucins [42]. We are currently addressing whether expression and/or localization glycosyltransferases involved in Tn antigen synthesis are affected by the overexpression of BRAF^V600E^.

In summary, we here propose a model in which activating BRAF mutations, and possibly other oncogenic alterations that activate the MAPK pathway, lead to an altered tumor cell glycosylation profile and enhanced expression of MGL ligands, presumably the Tn antigen. These aberrant glycans on tumor cells may have the ability to suppress anti-tumor immune responses through activation of the MGL receptor on (lymph node) DCs. Our findings are of particular interest in light of recent advances in immunotherapeutic treatment options of cancer patients, which focus on strategies that aim to reactivate the anti-tumor immune response by blocking molecular mechanisms that lead to immune suppression [43]. As such, the present study emphasizes the need to further investigate the direct impact of BRAF and other mutations in the MAPK-pathway on tumor cell glycosylation and immune evasion in CRC patients.

## MATERIALS AND METHODS

### Patients and tissue microarrays

Generation of tissue microarrays (TMAs) and clinical data of patients were published previously [23, 24]. In brief, formalin-fixed paraffin-embedded tissue blocks of CRC resection specimens of 386 stage II/III colon cancer patients were used for punching three core biopsies with a diameter of 0.6 mm from the central and the peripheral part of the tumor. Core biopsies were transferred into recipient paraffin blocks. Patients who were lost to follow-up or deceased within 3 months after surgery were excluded from further analysis. Patient characteristics are listed in Table 1.

### Immunohistochemistry

For MGL ligand expression, TMAs containing paraffin-embedded tissue material were deparaffinized, blocked with rabbit serum, incubated for 2 hours with MGL-mouse Fc (mFc) [44] at 37°C, washed, then incubated for 30 minutes with peroxidase-coupled rabbit-anti-mouse-IgG (DAKO, Heverlee, Belgium), followed by DAB (DAKO) and haematoxylin staining. A similar analysis was performed on paraffin-embedded tissue material of a previously described inducible BRAF^V600E^ mouse model [27]. Specificity of MGL-Fc binding was determined in the presence of 100 mM GalNAc monosaccharides.

### TMA analysis method

Stained sections were automatically scanned with a digital pathology system (Mirax slide Scanner system, 3DHISTECH, Budapest, Hungary), equipped with a 20x objective with a numerical aperture of 0.75 and a Sony DFW-X710 Fire Wire 1/3-inch type progressive SCAN IT CCD (pixel size 4.65 × 4.65 μm). Scoring was performed using dedicated TMA scoring software (v1.14.25.1, 3DHISTECH Ltd., Budapest, Hungary) on computer monitors calibrated using Spyder2PRO software (v1.0–16, Pantone Colorvision, Regensdorf, Switzerland). A chart with visual analogue scales of staining patterns was used to facilitate scoring. MGL-mFc staining in the cytoplasm of epithelial tumor cells, taken from the center or peripheral areas of tumor, was scored for staining intensity (negative, weak, moderate or strong) and frequency (0–25%, 26–50%, 51–75% or 76–100% of positive tumor cells). As no major differences were found between the center or the peripheral edge of the tumor, the average staining of all biopsies from one patient was used for the analysis.

### Statistical analysis

Statistical analysis was performed with SPSS 20.0 and R software (R Foundation for Statistical Computing). Optimal cut-off for dichotomizing scores into categories of low and high MGL-mFc binding was assessed by performing a receiver–operating characteristic (ROC) curve analysis [45, 46] of the intensity scores with 5-year survival as outcome of interest, followed by an identical analysis to further optimize the cut-off for frequency scores. Based on this analysis, the optimal cut-off was the cut-off stratifying staining frequency higher than 25% of cells with intensity scores moderate and strong as high MGL binding and all other scores as low MGL binding. Disease-free survival (DFS) and CRC-specific survival (CSS) were displayed in Kaplan-Meier curves and the hazard rate ratio (HRR) was calculated by Cox regression with survival as outcome. Multivariate Cox regression analysis for the stage II/III cohort was performed by combining MGL binding, age, location of tumor, chemotherapy, MSI status, tumor stage (T), disease stage, differentiation grade and angioinvasion. Levels of correlations were calculated using Pearson's Chi-square test. Differences were considered significant when *P* < 0.05.

### Cell culture and reagents

The colorectal cancer cell lines Colo320, SW1398, Colo205, HT29 and RKO were maintained in DMEM supplemented with 10% FBS and antibiotics. KM12 was maintained in RPMI 1640 supplemented with 10% FBS, glutamine and antibiotics. Cell lines were analyzed for mutations in *KRAS* exons 1 and 2, *BRAF* exon 15 and *EGFR* exons 18–21 by High Resolution Melting (HRM) and sequencing. All cell lines were routinely tested for mycoplasm infection. 5 × 10^5^ cells were seeded 24 hours before treatment with either the BRAF mutant inhibitors PLX4032 (Vemurafenib) or PLX4720 (Selleck Chemicals) or the MEK inhibitor U0126 (Invivogen) for the indicated time points and concentrations.

### Lectin flow cytometry

For the analysis of MGL ligand expression the previously described MGL-Fc [12] was used. FITC-labeled anti-human IgG-Fc antibody (Jackson Immunoresearch, Suffolk, UK) was used to detect binding of MGL-Fc by flow cytometry. Biotinylated lectin *Helix pomatia* agglutinin (HPA) was obtained from Sigma–Aldrich (st. Louis, MO). Extracellular MGL ligand expression was analyzed by flow cytometry as reported before [44]. Binding of HPA (5 μg/ml) was analyzed by incubating cells for 30 min at 37°C with the lectin, followed by staining with Alexa-488 labeled streptavidin (Molecular Probes, Waltham, MA) and analyzed on a FACSCalibur (BD Biosciences, Franklin Lakes, New Jersey). Cells were selected based on forward-scatter and side-scatter patterns and exclusion of dead cells by 7-amino-actinomycin D (Molecular Probes) staining. Intracellular phosphorylated-ERK1/2 levels were analyzed by fixing with 2% paraformaldehyde and permeabilizing with 90% methanol at 4°C, followed by incubation for 1 hour at room temperature with FITC-coupled rabbit-anti-phospho-ERK1/2 (Thr202/Tyr204) (Cell Signaling), followed by analysis on a FACSCalibur.

## SUPPLEMENTARY FIGURES


