# Supplementary material for: MGL ligand expression is correlated to BRAF mutation and associated with poor survival of stage III colon cancer patients
Source: Oncotarget. 2015 Jul 2;6(28):26278–90. doi: 10.18632/oncotarget.4495 (PMC4694901; doi:10.18632/oncotarget.4495)
Supplement: Supplementary file 1 [file oncotarget-06-26278-s001.pdf]

SUPPLEMENTARY FIGURES

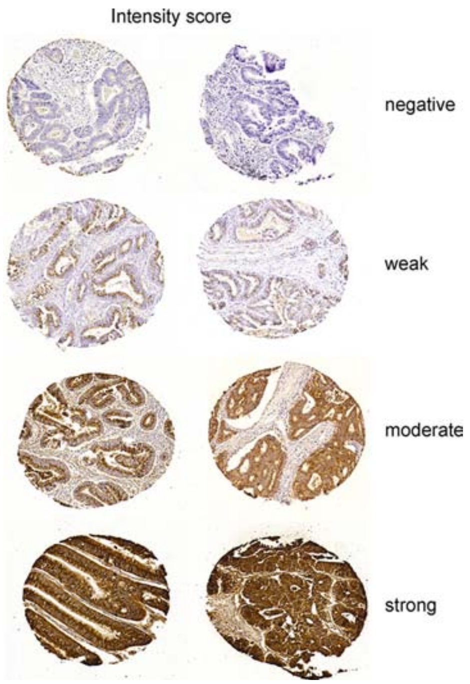

Supplementary Figure S1: Expression patterns of MGL-ligands in CRC. Colorectal cancer tissue microarray cores were stained for MGL-mFc and scored for intensity and frequency of staining in the cytoplasm of the tumor cells. Two examples are shown for respectively negative, weak, moderate and strong intensity staining.

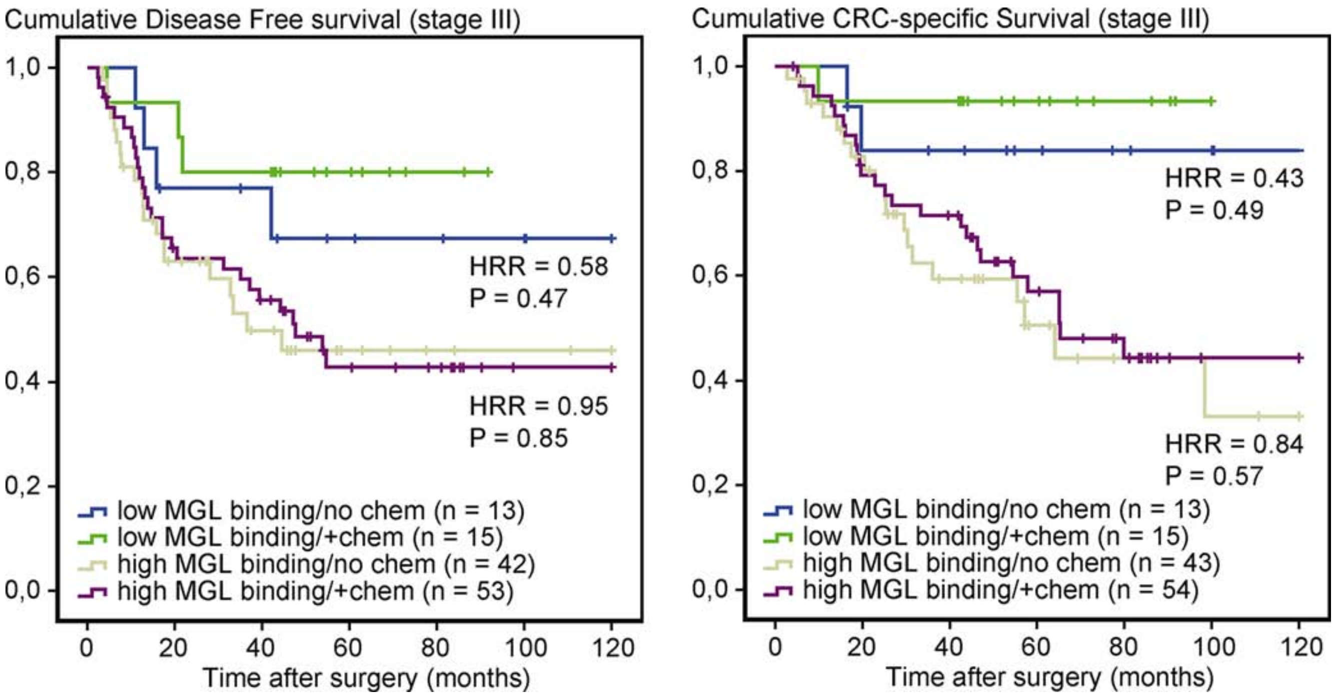

Supplementary Figure S2: MGL-mFc binding is independent of chemotherapy in stage III CRC patients. Kaplan-Meier curves of DFS and CSS curves in stage III CRC patients, stratified for adjuvant chemotherapy (no chemo/+chemo) and MGL-binding. Patients deceased within 3 months after surgery were excluded from the analysis. Hazard Risk Ratio and Significance (*p*-value) between chemo or no chemo within low or high MGL-binding group were determined by Cox regression analysis.
